# Herbal medicine and its impact on the gut microbiota in colorectal cancer

**DOI:** 10.3389/fcimb.2023.1096008

**Published:** 2023-07-04

**Authors:** Fan Bu, Yifeng Tu, Ziang Wan, Shiliang Tu

**Affiliations:** ^1^Department of Colorectal Surgery, Zhejiang Provincial People's Hospital, Affiliated People's Hospital of Hangzhou Medical College, Hangzhou, Zhejiang, China; ^2^The Second Affiliated College of Zhejiang Chinese Medical University, Hangzhou, Zhejiang, China

**Keywords:** microbial metabolism, gut microbiota, herbal medicine, colorectal cancer, bile acids

## Abstract

It is well-established that there are trillions of gut microbiota (GM) in the human gut. GM and its metabolites can reportedly cause cancer by causing abnormal immune responses. With the development of sequencing technology and the application of germ-free models in recent years, significant inroads have been achieved in research on GM and microbiota-related metabolites. Accordingly, the role and mechanism of GM in colorectal cancer (CRC) development have been gradually revealed. Traditional Chinese medicine (TCM) represents an important source of natural medicines and herbal products, with huge potential as anti-CRC agents. The potential application of TCM to target gut microbes for the treatment of colorectal cancer represents an exciting area of investigation.

## Introduction

Colorectal cancer (CRC) results from malignant transformation of intestinal epithelial mucosa mediated by various pathogenic factors. With lifestyle changes associated with the fast pace of living nowadays, the morbidity and mortality rates attributed to CRC have been increasing worldwide. Although the exact cause of CRC remains unclear, CRC development and progression have been linked to inheritance, immunity, environment, dietary habits, and lifestyle. Studies have shown that obesity, smoking and depression are risk factors for the development of CRC. In addition, individuals with inflammatory bowel disease (IBD) are associated with an increased incidence of colitis-associated cancer (CAC). Chronic inflammation is considered to be an essential initiating factor for the occurrence of CRC. The homeostasis of the intestinal microenvironment is essential to maintain the normal function of intestinal epithelial mucosa and inhibit the occurrence and development of CRC. Therefore, exploring the abnormal molecular mechanisms in the microenvironment of CRC and finding effective therapeutic targets are significant for preventing and treating this patient population.

With the rapid development and wide application of microbiota sequencing technology and metabolomics technology, researchers can detect microbes and their metabolites in the gut to understand the distribution of microbiota and the changes of metabolites in the gut microenvironment. Accordingly, we have gained a deeper understanding of gut microbiota. Importantly, the gut microbiota and its metabolites play a key role in maintaining intestinal homeostasis and inhibiting the occurrence of CRC. According to a recent study, the intestinal microbiota might act as the driving factor for the development of intestinal cancer. A “driver-passenger” pattern was also proposed, whereby the original intestinal bacteria (driving factor) attack the intestine, leading to DNA damage to cause CRC. Next, tumors cause changes in the intestinal microecology, which is conducive to the proliferation of conditioned bacteria (bacterial passengers) (Marchesi et al., 2016). A study transplanted gut microbiota (GM) from CRC patients and healthy humans into antibiotic-treated mice, and the mice were molded with azoxymethane (AOM). The results showed that significantly higher proportions of conventional mice that were fed stools from CRC individuals developed high-grade dysplasia (P<0.05) and macroscopic polyps (P<0.01) compared to mice fed stools from healthy controls. In addition, real-time Polymerase Chain Reaction (PCR) revealed the upregulation of genes involved in cell proliferation, stemness, apoptosis, angiogenesis, invasiveness, and metastasis in mice fed stools from CRC patients. Recent data suggest that in addition to the GM itself, its metabolites, such as short-chain fatty acids (SCFAs) such as acetate, propionate, and butyrate, can suppress inflammation and cancer, while secondary bile acids promote cancer. Dietary intake yields an important effect on the gut environment, much of which is mediated by the metabolic activities of the intestinal microbiota on dietary compounds. Indeed, different microbial metabolites can potentially promote and protect against CRC.

Herbal medicine has been in use for thousands of years in China and reportedly exerts a good therapeutic effect on many diseases via its multi-targeting properties. Although the effectiveness of herbal medicine has been established, the mechanism underlying its effects on the human body remains unclear. Orally administered herbal medicine decoction can interact with the GM and play an important role in maintaining intestinal barrier integrity and intestinal microenvironment homeostasis. Herbal medicine is widely thought to have beneficial biogenic effects on GM, playing an important therapeutic role by regulating the population, distribution and metabolites of intestinal microbiota to promote its probiotic function. The potential value of TCM in CRC warrants further investigation. In this review, we aimed to provide a plausible mechanistic basis for the interactions between herbal medicines and GM and the therapeutic benefits of this interaction in CRC.

## Relationship between GM and CRC

It is estimated that the total amount of GM genes is 100 times that of human genes (Komaroff, 2018). GM serves several functions in host metabolism, such as promoting immunity and protecting the intestinal barrier (Shin and Kim, 2018; Bender, 2020). Intestinal dysbiosis reduces the function of the intestinal mucosal barrier and enhances bacterial translocation, leading to inflammation and infection. Since the 1970s, germ-free mice have been used to demonstrate that alterations in the gut microbiota play an important role in the development of CRC (Reddy et al., 1976; Drewes et al., 2022; Zhao et al., 2022). Recent studies have shown that GM is a critical environmental factor in regulating body metabolism, contributing to the occurrence and development of CRC. During the early stages of CRC, the microbiota is characterized by decreased abundance of commensal bacteria that produce anti-inflammatory SCFAs, such as *Lactobacillus*, *Bifidobacterium*, and *Clostridium*, and markers of intestinal epithelial damage (such as diamine oxide, D-lactic acid and LPS). In addition, studies have shown that *Fusobacterium nucleatum* (*F. nucleatum*) and *Escherichia coli* (*Escherichia coli pks+*) are positively correlated with metabolites such as diamine oxide, D-lactic acid, and LPS (Liu et al., 2020).

Alterations in GM in CRC can be divided into three different patterns. in that first model, the abundance of some pro-inflammatory bacteria, such as *Fusobacterium nucleatum*, *Peptostreptococcus anaerobius* and *Peptostreptococcus stomatis*, increase continuously from stage 0 to progressive stage. In CRC stage I/II and stage III/IV, *Parvimonas micra*., *P. anaerobius*, *P. stomatis*, and *P. micra*, while their levels decreased after tumor resection, suggesting that these species may simply adapt to the cancer microenvironment but not participate in the occurrence of CRC. In the second pattern, the abundance of *Atopobium parvulum* and *Actinomyces odontolyticus* increased significantly in stage 0 of multiple polypoid adenomas, but did not increase in advanced and advanced stages. The third pattern, anti-inflammatory micro-organisms or certain probiotics, such as butyrate-producing bacteria (*Lachnospira multipara* and *Eubacterium eligens*) and *Bifidobacterium longum*, decreases as CRC progresses. The changes of intestinal microbiota composition in different stages of CRC are also different.

## Potential causative bacteria and their metabolic pathways in CRC

Following the discovery that *Helicobacter pylori* increase the risk of gastric cancer (Caruso and Fucci, 1990), other species colonizing the gastrointestinal tract have been investigated for potential cancer-inducing properties. In a recent study, germ-free mice and conventional mice with AOM were fed stool samples from patients with CRC and healthy individuals. It was found that stool from patients with CRC increased the number of polyps, intestinal dysplasia and proliferation, markers of inflammation, and proportions of Th1 and Th17 cells in the colon, compared with stool from individuals without CRC. This study provides evidence that the fecal microbiota from patients with CRC can promote tumorigenesis in germ-free mice and mice (Wong et al., 2017).

*Escherichia coli* is an opportunistic pathogen that participates in the digestion and synthesis of vitamin K2 under normal circumstances but becomes pathogenic when the body’s immunity declines. Strains of *Escherichia coli* (*E. coli*) have been identified as a potential risk factor for CRC. *E. coli* was found to colonize cancer lesions and neighboring epithelium, suggesting they are the only cultivable organisms in close contact with the diseased site. Analysis of mucosa-associated *E. coli* from colon cancer showed that 66% of biopsies from CRC were positive for *E. coli* strains by bacterial culture and *B2 E. coli strain 11G5* isolated from colon cancer can persist in the gut, and induce colon inflammation, epithelial damage and cell proliferation (Raisch et al., 2014). In another study, IL-10 double knockout mice were treated with the carcinogen AOM, and the animals were raised germ-free before they were mono-colonized with *E. coli*, suggesting the carcinogenic effect of the *E. Coli strain NC101* (Arthur et al., 2012). In a mouse model of CRC, colibactin-producing *pks+ E. coli* was found to induce DNA double-strand breaks and mutations and promote tumor development. Recent research using mouse colon organoids demonstrated that *pks+E. coli* could promote the malignant transformation of colon organoids *in vitro*, emphasizing the relevance of copy number variation in driving the early development and progression of colon cancer (Iftekhar et al., 2021).

Recent studies have reported that the anaerobic gram‐negative oral commensal bacterium, *F. nucleatum*, plays a significant role in CRC. Cytochrome P450 monooxygenase (mainly CYP2J2) and its mediated product 12,13-EpOME were upregulated in tumor cells from CRC patients and mouse models of CRC, which activated epithelial-mesenchymal transition (EMT) *in vitro* and promoted CRC cell invasion and migration *in vivo*. Fecal *F. nucleatum* levels were positively correlated with serum 12,13-EpOME levels in CRC patients. *F. nucleatum* may promote CYP2J2 transcription by activating TLR4/AKT signaling, downregulating Keap1 and increasing NRF2 (Kong et al., 2021). Another research suggested that *F. nucleatum* binds to the T-cell inhibitory immune receptor of natural killer cells (NK) through another adhesin, Fap2 (Gur et al., 2015). *F. nucleatum* has also been shown to activate the cellular survival mechanism, autophagy, through TLR4 receptors located on CRC cell surfaces in response to oxaliplatin chemotherapy (Yu et al., 2017). In addition, *F. nucleatum* was found to yield an antagonistic effect on probiotics and was involved in establishing a multispecies microbial community in the large intestine. The extracellular protein secreted by *F. nucleatum* exhibited strong bacteriostatic activity against the probiotics *F. nucleatum*, *Bifidobacterium*, and *Lactobacillus*. Inhibiting the anti-inflammatory effect of probiotics might be a new reason for the *Faecalibacterium prausnitzii* being involved in CRC. A combination of *F. nucleatum*/*Bifidobacterium* and *F. nucleatum*/*Faecalibacterium prausnitzii* had superior sensitivity and specificity in detecting stage I CRC. Indeed, developing microbial ratio detection approaches has huge value for large-scale screening of early CRC (Guo et al., 2018).

There are 2 classes of *Bacteroides fragilis* distinguished by their ability to secrete a zinc-dependent metalloprotease toxin, *Bacteroides fragilis toxin* (BFT). Strains that do not secrete BFT are called nontoxigenic *Bacteroides fragilis* (NTBF), and those that do are referred to as enterotoxigenic *Bacteroides fragilis* (ETBF) (Wu et al., 2007). The ETBF can reportedly cause gastrointestinal (GI) inflammation and inhabit biofilms coating human CRC. Intestinal inflammation is mediated by BFT, the only known virulence factor of ETBF. BFT-induced increased barrier permeability has been reported to correlate with cleavage of the intercellular adhesion protein of the zonula adherens, E-cadherin. E-cadherin cleavage yields multiple potentially procarcinogenic triggers, including Wnt signaling, CEC proliferation, and epithelial barrier disruption that promote mucosal inflammation (Grivennikov et al., 2012). Recent experimental evidence demonstrating that ETBF-driven colitis promotes colon tumorigenesis has generated interest in the potential contribution of ETBF to human colon carcinogenesis. Other studies revealed additional mechanisms; BFT induces the antiapoptotic protein cIAP2 and the polyamine catalyst spermine oxidase, which triggers reactive oxygen species production, DNA damage, and cell proliferation (Kim et al., 2008). BFT also induces rapid DNA damage in colon epithelial cells *in vivo*, as detected by activation of the H2A histone family, member X, an initiator of DNA repair (Goodwin et al., 2011). A recent study showed that in multiple intestinal neoplasia (Min) mice, ETBF-induced inflammatory colitis leads to the development of colon cancer within 4 weeks. To sum up, cleavage of E-cadherin, activation of NF-κB (with antiapoptotic effects), increased polyamine metabolism, and induction of DNA damage within the colonic epithelium are key BFT oncogenic mechanisms identified to date in studies.

*Enterococcus faecalis* is the first colonizer of the human GI tract and has a major impact on intestinal immune development in the very early stages of life. Enterococcus faecalis produces substantial extracellular superoxide and derivative reactive oxygen species and hydroxyl radicals through autoxidation of membrane-associated demethylmenaquinone. These oxidants may cause chromosomal instability associated with sporadic adenomatous polyps and CRC. Enterococcus faecalis has also been demonstrated to produce metalloprotease that can directly compromise the intestinal epithelial barrier and induce inflammation (Huycke et al., 2002). In contrast, in another study, 5-week-old male and female Apc mutant Min mice were fed diets containing heat-killed *Enterococcus faecalis strain EC-12* (*EC-12*) for 8 weeks. A 4.3% decrease in the number of polyps was observed in males vs. 30.9% in females. Moreover, heat-killed *EC-12* reduced c-Myc and cyclin D1 mRNA expression levels in intestinal polyps (Miyamoto et al., 2017). *Enterococcus faecalis* reportedly become pathogenic only during major environmental alterations, such as during the appearance of cytokines and mucins or changes in oxygen tension (de Almeida et al., 2018).

A study showed that compared with healthy subjects, the feces and colon tissue of CRC patients were significantly enriched in *P. micromonas* (*P. micra*), which was associated with poor prognosis of CRC patients. In addition, oral administration of *P. micra* promoted colorectal tumorigenesis in Apc^min/+^ mice and colonic epithelial cell proliferation in C57BL/6 mice and germ-free mice. In another microflora analysis, isolation and culture combined with experimental data showed that the toxin-producing strains of *Clostridium* difficile in the mucosal sera of CRC patients could induce colon tumors in mice, and its tumor-promoting effect depended on the continuous colonization of the strains and the production of the toxin TcdB. An increasing body of evidence suggests that *Peptostreptococcus anaerobius* (Tsoi et al., 2017), *Parvimonas micra* (Zhao et al., 2022), *Salmonella* (Mughini-Gras et al., 2018) and other gut microbiota members can also influence the development of CRC.

## Potential beneficial bacteria and their metabolic pathways in CRC

Interestingly, some gut microbiota members yield a pathogenic effect in CRC, while others play a preventive role. Probiotic bacteria confer a health benefit on the host when administered adequately. A review retrieved 21 clinical trials involving 1831 patients undergoing elective colorectal surgery. According to the study results, probiotics could significantly decrease inflammatory factors, chemotherapy side effects, severe diarrhea, postoperative infectious complications, and duration of antibiotic therapy (Darbandi et al., 2020).

Some reports have indicated that lactic acid bacteria (LAB) isolated from different sources possess certain anticancer activity and properties. Intriguingly, lactic acid bacteria have antitumor properties that inactivate or inhibit carcinogenic compounds in the gastrointestinal tract, stimulate the immune response, and reduce the enzymatic activity of β-glucuronidase, azoreductase, and nitroreductase. A pivotal study on a murine model of CRC induced by AOM and Dextran Sulfate Sodium (DSS) revealed significantly alleviated inflammation and tumor development in mice treated with *Lactobacillus* compared to the CRC mice. *Lactobacillus* species inhibited tumor growth, and its anticancer activity was at least partially mediated by suppressing the Wnt/β-catenin pathway (Ghanavati et al., 2020). Another research used faecal shotgun metagenomic sequencing to show the depletion of *Lactobacillus gallinarum* in patients with CRC. In addition, it was suggested that *Lactobacillus gallinarum* protects against intestinal tumorigenesis by producing protective metabolites (indole-3-lactic acid) that can promote apoptosis of CRC cells (Sugimura et al., 2021). *Lactobacillus casei BL23(L. casei BL23)* is a probiotic strain known for its anti-inflammatory and anticancer properties. *L. casei BL23* significantly protected mice against CRC development and reduced histological scores and proliferative index values. *L. casei BL23* may exert an antitumor effect mediated through the downregulation of IL-22 cytokine and upregulation of caspase-7, caspase-9, and Bik (Jacouton et al., 2017). *Lactobacillus paracasei* (*L. paracasei*) is a lactic acid bacteria strain isolated from feta-type cheese that has huge prospects for application for CRC treatment. Current evidence suggests that *L. paracasei* induce CRC cells apoptosis and inhibit their proliferation by regulating the expression of specific Bcl-2 family proteins, producing reactive oxygen species (ROS), inducing the translocation of calreticulin (CRT) and changing the cell cycle (Chondrou et al., 2018; Nozari et al., 2019). Another research showed that the extracellular vesicles of *L. paracasei* (LpEVs) were taken up by CRC cells and inhibited their proliferation, migration, invasion and apoptosis. Transcriptome sequencing analysis revealed that LpEVs significantly inhibited the phosphorylation level of 3-phosphoinositide-dependent protein kinase-1 (PDK1) and AKT in CRC cells and reduced the expression of Bcl-2 protein (Shi et al., 2021).

Gianotti et al. conducted a randomized placebo-controlled clinical trial demonstrating the beneficial effects of two probiotic LAB strains, *Bifidobacterium longum BB536* and *Lactobacillus johnsonii La1*, in this patient group (Gianotti et al., 2010). *B. bifidum* has been reported to yield anti-proliferative and protective effects against preneoplastic lesions in animal models of colorectal carcinogenesis (Mohania et al., 2014). Interestingly, some species of *Bifidobacteria* can decrease carcinogen-induced DNA damage, preneoplastic lesions, and tumors in the colon of rats (Oberreuther-Moschner et al., 2004). It has been reported that whole peptidoglycan, a metabolite produced by *Bifidobacterium*, could activate macrophages to produce large amounts of cytotoxic molecules, including TNF-α, IL-6, and IL-12 (Wang et al., 2001). Another study investigated the effects of secretion metabolites from different *Bifidobacteria* species on CRC cell lines. The results showed that the anticancer activity of *Bifidobacteria* was mediated by the downregulation and upregulation of antiapoptotic and proapoptotic genes, respectively (Faghfoori et al., 2021).

Recent research found that *Streptococcus thermophilus* (*S thermophilus*) was depleted in patients with CRC. The antitumor effects of *S thermophilus* were assessed in 2 murine models of intestinal tumorigenesis. Oral gavage of *S thermophilus* significantly reduced tumor formation in Apc^min/+^ and azoxymethane-injected mice. The tumor-suppressive effect of *S thermophilus* was likely mediated by the secretion of β-galactosidase (Li et al., 2021).

A recent study used an ex vivo model of the human colon to investigate the impact of the bacteriocin-producing *S. salivarius DPC6993* on *F. nucleatum*, a gut pathogen associated with CRC. Results indicated that *S. salivarius DPC6993* could suppress the growth of the CRC-associated bacteria within the gut environment and exert minimal impact on the surrounding GM. As biotherapeutics for suppressing the growth of *F. nucleatum* in the human gut, these bacteriocin-producing strains can potentially reduce the risk of CRC development. A marked reduction in butyrate-producing bacteria has been observed in patients with CRC. A study found that *Clostridium butyricum* (*C. butyricum*) (one of the commonly used butyrate-producing bacteria) significantly inhibited high-fat diet (HFD)-induced intestinal tumor development in Apc^min/+^ mice. Moreover, intestinal tumor cells treated with *C. butyricum* exhibited decreased proliferation and increased apoptosis. It was concluded that *C. butyricum* could inhibit intestinal tumor development by modulating Wnt signaling and GM (Chen et al., 2020).

Another study examined the effects of butyrate-producing *Butyricicoccus pullicaecorum* (*B. pullicaecorum*) on mice with 1,2-dimethylhydrazine (DMH)-induced CRC. Administration of *B. pullicaecorum* reduced symptoms of CRC by increasing levels of expression of proteins and mRNAs in the 5-limb solute carrier family 8 and the G 43 protein-coupled receptor (Chang et al., 2020).

## Microbiota-associated metabolites and CRC

GM can ferment food residues and produce many metabolites, which regulate biological functions in the human body. The main fermentation products in healthy adults are gases and organic acids, especially the three SCFAs acetic, propionic and butyric acids. SCFAs are by-products of dietary fiber in the gut during fermentation and are the most important energy source of the colonic epithelial lining.

### Short-chain fatty acids

The GM of CRC patients can increase pathogenic bacterial abundance and reduce SCFA-producing bacteria and SCFA production. The most common SCFA-producing probiotics are *Clostridium butyricum*, *Bifidobacterium*, *Lactobacillus rhamnosus*, *Streptococcus thermophilus*, *Lactobacillus reuteri*, *Lactobacillus casei*, and *Lactobacillus acidophilus*. SCFAs promote intestinal homeostasis and epithelial integrity by improving intestinal mucosal growth, colonic blood flow, and water and electrolyte absorption (Macia et al., 2015). *In vitro* and *in vivo* studies have shown that SCFAs can ameliorate the LPS-induced intestinal barrier disruption by suppressing NLRP3 inflammasome and autophagy by inhibiting ROS production (Feng et al., 2018). SCFAs are key regulators of immune function with immunomodulatory capabilities on various immune cells, including Treg cells, macrophages, and B cells. The host immune system mainly senses and recognizes gut bacterial metabolites by activating SCFA-GPCRs or inhibiting histone deacetylase (HDAC), thereby influencing the host immune response. Propionate in SCFAs promotes its inhibitory activity by inhibiting HDAC through the GPR43 signaling pathway, preventing the occurrence of T cell-induced colitis (Smith et al., 2013).

### Bile acid

Epidemiological data suggest that the incidence of CRC is higher in patients with higher fecal bile acid concentrations. It should be borne in mind that not all bile acids promote CRC. Many experiments have shown that high concentrations of secondary bile acids (SBAs) play a role in tumor promotion during the development of CRC. A study revealed that the concentrations of deoxycholic acid (DCA), lithocholic acid (LCA), ursodeoxycholic acid (UDCA), and other indicators in the feces of CRC patients were higher than in the normal control group, while primary bile acids (cholalic acid

, chenodeoxycholine acid) levels were not different from the control group (Rider et al., 1984). It has been established that bile acids exert strong antimicrobial activities, as they damage bacterial cell membranes owing to their amphipathic properties and are, therefore, likely to modify the composition of the GM. Rats fed a diet supplemented with deoxycholic acid showed decreased production of SCFAs and major changes in the microbiota composition, with a relative increase in Gamma-proteobacteria and certain *Firmicutes* at the expense of *Bacteroidetes*.

Animal experiments substantiated that SBAs have more potent effects than primary bile acids (PBAs), especially in promoting tumorigenesis and as auxiliary carcinogens (Imray et al., 1992). A high-fat diet and gallbladder disease can increase the content of SBAs in the intestinal cavity, especially DCA levels, thereby promoting the occurrence and development of CRC. The molecular mechanisms that mediate the cytotoxic effects of bile acids are complex. SBAs are more hydrophobic and thus more potent at disrupting cell membranes, which is likely to lead to the generation of ROS via the activation of membrane-associated proteins such as NAD(P)H oxidases and phospholipase A2 (Majer et al., 2014). In contrast, some bile acids seem to counteract the cytotoxic effects of others. In this respect, ursodeoxycholic acid can inhibit the production of ROS and protect cells from the cytotoxic effects of deoxycholic acid (Lee et al., 2013).

### Polyamines

Polyamines are derived from endogenous synthesis and GM metabolism of amino acids (Tofalo et al., 2019). Altered polyamine levels are related to cancer. In cancer cells, polyamine metabolism is frequently dysregulated primarily through the upregulation of the polyamine biosynthetic enzymes, leading to elevated polyamine levels necessary for malignant transformation and tumor progression. Polyamine levels were found to increase throughout tumor development in an APC^Min/+^ murine CRC model. Additionally, biofilm formation in colon cancer increases polyamine metabolites, suggesting polyamines produced by biofilm bacteria or the host enhance tumor development (Holbert et al., 2022). Recent research demonstrated elevated amino acids and polyamines levels in CRC patients, especially cadaverine and putrescine (Hanus et al., 2021). Spermine synthase (SMS), a polyamine biosynthetic enzyme, is reportedly overexpressed in CRC. In a recent study, disruption of the SMS gene in CRC cells was found to alter polyamine metabolism by dramatically reducing the levels of spermine and putrescine but producing excessive levels of spermidine (Guo et al., 2020). These studies indicate that SMS overexpression is required to balance spermidine levels to facilitate CRC cell growth. SMS cooperates with MYC to maintain CRC cell survival via distinct pathways that converge to repress the expression of the proapoptotic protein Bim. Combined inhibition of SMS and MYC signaling induces synergistic apoptosis and tumor regression.

In addition to the above-mentioned metabolite, other metabolites metabolized by intestinal flora, such as glycerophospholipids, tryptophan, trimethylamine oxide, etc., also contribute to CRC via their proinflammatory properties or stimulation of immune responses.

## Herbal extract, GM and CRC

Herbal medicine has been in use for thousands of years in China and has a good therapeutic effect against many diseases via its multi-targeting effects. Although the effectiveness of herbal medicine has been established, the mechanism underlying its effects on the human body remains unclear. Oral and enema herbal medicine can significantly affect the distribution and number of GM. Herbal medicine might have beneficial biogenic effects on GM, and it plays an important therapeutic role by regulating the population, distribution and metabolites of intestinal microbiota to promote its probiotic function in CRC. Both these compound formulas and a variety of products of these main constituent herbs, such as extracts, fractions, or single compounds, have demonstrated therapeutic benefits in CRC. In this section we will focus on herbal extracts.

The isoquinoline alkaloid berberine extracted from the TCM, Coptis chinensis, has been documented to have specific antibacterial activity. It has been shown that berberine significantly ameliorates CRC progression in Apc^min/+^ mice fed with an HFD, with reduced numbers of tumors and decreased inflammation in colon tissues (Wang et al., 2018). Treatment with berberine reversed the imbalance in intestinal microbiota caused by *F. nucleatum* colonization and was characterized by an increase in *Tenericutes* and *Verrucomicrobia*. At the genus level, some SCFA-producing bacteria increased in abundance (Zhang et al., 2013). SCFAs administration prevented tumor development and attenuated the colonic inflammation in a mouse model of CAC. It is widely acknowledged that GM produces SCFAs by fermentation of dietary fiber (Tian et al., 2018).

GM also ferments the non-digestible plant polysaccharides. Interestingly, polysaccharides, the predominant component of jujube fruit, have been shown to inhibit carcinogenesis in animal models. Research showed jujube polysaccharides (JP) could ward off colon cancer by ameliorating colitis cancer-induced gut dysbiosis. There was a significant decrease in *Firmicutes/Bacteroidetes* post-JP treatment. Jujube could restore the GM profile altered by AOM/DSS, indicating the potential of jujube polysaccharides as promising prebiotic candidates for the prevention and treatment of CRC (Ji et al., 2020). Another research showed Zizyphus jujuba cv. Muzao polysaccharides (ZMP) consumption prevented CRC mouse colon shortening, decreased their mortality, reduced proinflammatory cytokines, and increased the concentration of total SCFAs and the abundance of *Bifidobacterium*, *Bacteroides* and *Lactobacillus* (Ji et al., 2019).

Carboxymethylated pachyman (CMP) is a polysaccharide modified from the structure of pachyman isolated from Poria cocos. Recent studies have shown that CMP exhibits immune regulatory, anti-inflammatory and antioxidant activities. CMP combined with 5-fluorouracil (5-FU) reversed intestinal shortening (p < 0.01) and alleviated 5-FU-induced colon injury (p < 0.001) via suppression of ROS production. Besides, an increase in the levels of CAT, GSH-Px and GSH and a decrease in NF-κB, p-p38 and Bax expression were observed. More importantly, CMP had a significant impact against GM disorders produced by 5-fluoro-2, 4(1H,3H)pyrimidinedione (5-FU) by increasing the proportion of *Bacteroidetes*, *Lactobacilli*, butyric acid-producing and acetic acid-producing bacteria and restoring the GM diversity (Wang et al., 2018). 5-FU plays an important role in palliative and adjuvant systemic chemotherapy for CRC. Over the past decades, various regulatory strategies, such as the implementation of 5-FU-based combination regimens and 5-FU pro-drugs, have been developed and tested to enhance antineoplastic activity and reduce clinical resistance (Vodenkova et al., 2020). Emerging evidence suggests combining conventional chemotherapy with some natural dietary polyphenols for CRC treatment can significantly enhance the chemotherapeutic effect. In addition to CMP, resveratrol and curcumin enhanced the efficacy of 5-FU (Shakibaei et al., 2014; Buhrmann et al., 2015; McFadden et al., 2015). Moreover, resveratrol can be used with other chemotherapeutic agents to overcome drug resistance since it downregulates multidrug resistance protein 1 by blocking the activation of the NF-κB signaling pathway and inhibiting the transcriptional activity of cAMP response elements (Wang et al., 2015). Another study revealed the efficacy of polysaccharides isolated from Nostoc commune Vaucher (NVPS) against CAC tumorigenesis in mice treated with AOM/DSS. The treatment with NVPS significantly decreased the number and size of tumors and reduced the incidence of intestinal tumors. Moreover, the abundance of SCFA-producing genera, including butyric acid-producing genera (*Butyricicoccus*, *Butyrivibrio* and *Butyricimonas*) and acetic acid-producing genera *(Lachnospiraceae UCG 001*, *Lachnospiraceae UCG 006*, and *Blautia*), was significantly enriched following the NVPS treatment. These compositional alterations induced by NVPS were associated with suppressed colonic inflammation and carcinogenesis (Guo and Li, 2019). The study on the regulation of the intestinal microbiota and the inhibition of tumor cell proliferation by algae polysaccharides is of great importance to further explore the antitumor mechanism of algae polysaccharides. Research has shown that Nostoc flagelliforme capsule polysaccharides decreased the abundance of pathogenic bacteria and increased the abundance of probiotics positively correlated with SCFAs. Enteromorpha clathrata dietary polysaccharide and placine polysaccharides can promote tumor cell apoptosis while increasing the abundance of lactobacilli in the intestine, providing a theoretical basis for the future direction of regulating intestinal microbiota with seaweed polysaccharides in the treatment of tumors (Shang et al., 2018; Li et al., 2022).

A meta-analysis revealed that a high intake of certain dietary flavonoids is associated with decreased colon and rectal cancer risk. It has been shown that members of the GM significantly contribute to the metabolism of flavonoids, and flavonoids can alter the composition of the gut microbiome, highlighting that the microbiota plays a central role in the bioactivity of flavonoids (Li et al., 2018). Isoliquiritigenin (ISL), a flavonoid extracted from licorice, has shown antitumor efficacy. The levels of *Bacteroidetes* decreased and *Firmicutes* increased during CAC development. ISL reversed the imbalance at the phylum level, and the abundance of *Helicobacteraceae* increased after treatment with high-dose ISL, while *Lachnospiraceae* and *Rikenellaceae* decreased. At the genus level, ISL reduced the abundance of opportunistic pathogens (*Escherichia* and *Enterococcus*) and increased the levels of probiotics, particularly butyrate-producing bacteria (*Butyricicoccus*, *Clostridium*, and *Ruminococcus*). These results suggest a synergistic anticancer effect between ISL and GM (Wu et al., 2016).

The flavonoid is a common component found in many medicinal herbs. Apigenin can reportedly exhibit tumor suppression *in vitro* and *in vivo*. In a recent study, the size and number of tumors were reduced significantly in the apigenin treatment group. Fecal transplantation provided further evidence of apigenin-modulated GM exerting antitumor effects since Apigenin could not reduce the number or size of tumors when GM was depleted (Bian et al., 2020). Flavanols constitute a very complex group of flavonoids. Catechins, known as the major building blocks of tannins, are the most important representatives of flavanols. The structure of the GM of mice interfered with green tea extract epigallocatechin gallate (EGCG) remained relatively stable. Probiotics such as *Lactobacillus* and *Bifidobacterium* were increased in the EGCG+AOM/DSS group, and the relative abundance of *Lactobacillus* was significantly increased after long-term EGCG intervention. Its main mechanisms may include optimizing the combined GM to inhibit the growth of carcinogenic bacteria and the production of antitumor active substances (Wang et al., 2017). Berries are known to be a rich source of flavonoid compounds, especially anthocyanins. A recent research, six commonly consumed berries and their phenolic compounds, such as anthocyanins, flavonols, flavanols, ellagitannins, gallotannins, proanthocyanidins, and phenolic acids, were tested *in vitro* to evaluate their anticancer effects in HT-29/HCT116 colon cancer cells. Black raspberry and strawberry extracts showed the most significant proapoptotic effects against human cancer cells (Bouyahya et al., 2022). Another research showed that black raspberry (BRB) powder anthocyanins could modulate the composition of gut commensal microbiota, increase the abundance of probiotics such as *Eubacterium rectale*, *Faecalibacterium prausnitzii* and *Lactobacillus*, and changes in inflammation and the methylation status of the SFRP2 gene may play a central role in the chemoprevention of CRC (Chen et al., 2018).

Similar to other classes of flavonoids, the antitumor activity of the flavonol quercetin has been demonstrated against colon cancer cells both *in vitro* and *in vivo*. Quercetin can be metabolized and degraded into the SCFAs acetate and butyrate, associated with cytoprotective effects in the intestinal epithelium and protection against colon tumorigenesis (Li et al., 2018). Quercetin treatment reduced the abundance of *Proteobacteria*, which commonly blooms in colitis and during inflammation and in patients with IBD (Hughes et al., 2017). To date, no studies have demonstrated a positive effect of quercetin supplements on CRC risk in humans. In the polyp prevention trial, a high intake of flavonols, including quercetin, was associated with a significantly reduced risk of late-stage adenoma recurrence, while quercetin alone was not associated with adenoma recurrence (Bobe et al., 2008).

Gynostemma pentaphyllum (Gp) is a dietary herbal medicine. Gp saponins (GpS) were found to exhibit prebiotic and cancer-preventive properties through the modulation of GM in Apc^Min/+^ mice. One of the beneficial effects of GpS is its effectiveness in suppressing potentially harmful bacteria, such as sulfur-reducing bacteria and promoting SCFA-producing bacteria. It was found that inoculation with fecal materials from the GpS-treated Apc^Min/+^ mice effectively reduced the polyp burden in the untreated Apc^Min/+^ mice. Besides, a marked increase in the beneficial bacteria was detected in the feces of the FMT recipient Apc^Min/+^ mice. These bacteria include *Lactobacillus*, *Bifidobacterium* and *Clostridium cluster IV*, well-known probiotics (Liao et al., 2021).

Another research reported two of the most abundant compounds of GpS(ginsenosides Rb3 and Rd) for anti-CRC properties in Apc^Min/+^ mice. The anti-CRC properties correlated with the growth of beneficial bacteria such as *Bifidobacterium*, *Lactobacillus* and *Bacteroides acidifaciens*. In contrast, the abundance of cancer cachexia-associated bacteria, such as *Dysgonomonas* and *Helicobacter*, was significantly lower in Rb3/Rd-treated mice. Treatments of ginsenosides Rb3 and Rd exerted anticancer effects by holistically reinstating mucosal architecture, improving mucosal immunity, promoting beneficial bacteria, and downregulating cancer-cachexia-associated bacteria (Huang et al., 2017).

Panax quinquefolius L. is a commonly used herbal medicine, and ginsenosides are considered to be its bioactive constituents. The oral administration of ginseng (15 and 30 mg/kg/day) significantly suppressed AOM/DSS-induced colitis, as demonstrated by the disease activity index and colon tissue histology during the acute phase. Ginseng significantly suppressed AOM/DSS-induced tumor multiplicity during the chronic phase. Serum metabolomics data and the 16S rRNA data showed that after AOM/DSS, the microbiome community and serum metabolomics in the model group was significantly changed, and ginseng inhibited these changes. Oral ginseng significantly decreased AOM/DSS-induced colitis and colon carcinogenesis by inhibiting inflammatory cytokines, increasing the glutamine level and decreasing *Bacteroidetes* and *Verrucomicrobia* (Wang et al., 2016). In another example, zerumbone, the main component of Zingiber zerumbet, has been reported to have antibacterial, anti-inflammatory and antitumor activities and can reportedly reduce ETBF-induced, intestinal inflammation-related CRC by altering the IL-17, β-catenin, Stat3, and NF-κB pathways (Hwang et al., 2020).

## Traditional Chinese medicine compounds, GM and CRC

Chinese compounds are the material basis of TCM for the treatment of diseases. Each Chinese compounds contains hundreds of chemical molecules, and one compound is a combination of thousands of chemical molecules. How these molecules with diverse chemical structures interact with the complex biological regulatory network of the body, so as to realize the role of strengthening the body and strengthening the capital, exorcising evil spirits and dispelling diseases, has always been the scientific frontier and important issue constantly explored by modern Chinese pharmacists. Chinese compound formula is mainly taken orally. After the complex Chinese compound formula reach the intestinal tract, they will interact with the GM. Analysis the effects and relationship between the active molecules of natural medicine, GM and host will provide new evidence and ideas for the study of the efficacy and mechanism of Chinese medicine, and help to clarify the function and molecular mechanism of GM. Previous studies indicated that TCM yields potential anticancer effects in improving quality of life and therapeutic effect. However, little is known about the mechanism of the TCM formula in cancer prevention.

Gegen Qinlian decoction (GQD), a classical TCM formula, has been proven effective in treating ulcerative colitis (UC). A systemic pharmacological study revealed that combination therapy with GQD and anti-mouse PD-1 potently inhibited the growth of CT26 tumors in a xenograft model. GM analysis revealed that combination therapy with GQD and anti-mouse PD-1 significantly enriched for *Bacteroides_acidifaciens* and *Bacteroidales_S24-7_group*. Direct treatment with GQD and anti-mouse PD-1 increased the expression of IFN-γ, which is a critical factor in antitumor immunotherapy. The Chinese medicinal formula GQD, combined with PD-1 blockade-based immunotherapy, represents a novel therapeutic strategy for CRC patients (Lv et al., 2019). It has been reported that Yi-Yi-Fu-Zi-Bai-Jiang-San (YYFZBJS) treatment blocked tumor initiation and progression in Apc^Min/+^ mice with less change of body weight and increased immune function. The result determined the effect of YYFZBJS on the mRNA expression of T-bet, Gata3, ROR-γt, and Foxp3, which are considered as the master regulator of Helper T lymphocytes development and function in the immune system of mice. Moreover, diversity analysis of fecal samples demonstrated that YYFZBJS regulated animal’s natural GM, including *Bacteroides fragilis*, *Lachnospiraceae*, etc. Intestinal tumors from conventional and germ-free mice fed with stool from YYFZBJS volunteers were decreased. Overall, YYFZBJS represents a new potential drug option for treating CRC (Sui et al., 2020). In addition, Wu Mei Wan (WMW) has been used for clinically treating colitis with remarkable efficacy. Our previous research investigates the underlying mechanism of WMW in preventing CAC. WMW attenuates CAC by regulating the balance between “tumor-promoting bacteria “(*Bacteroidales_s24-7_group*) and “tumor-suppressing bacteria”(*Lachnospiraceae*).

**Figure 1:**
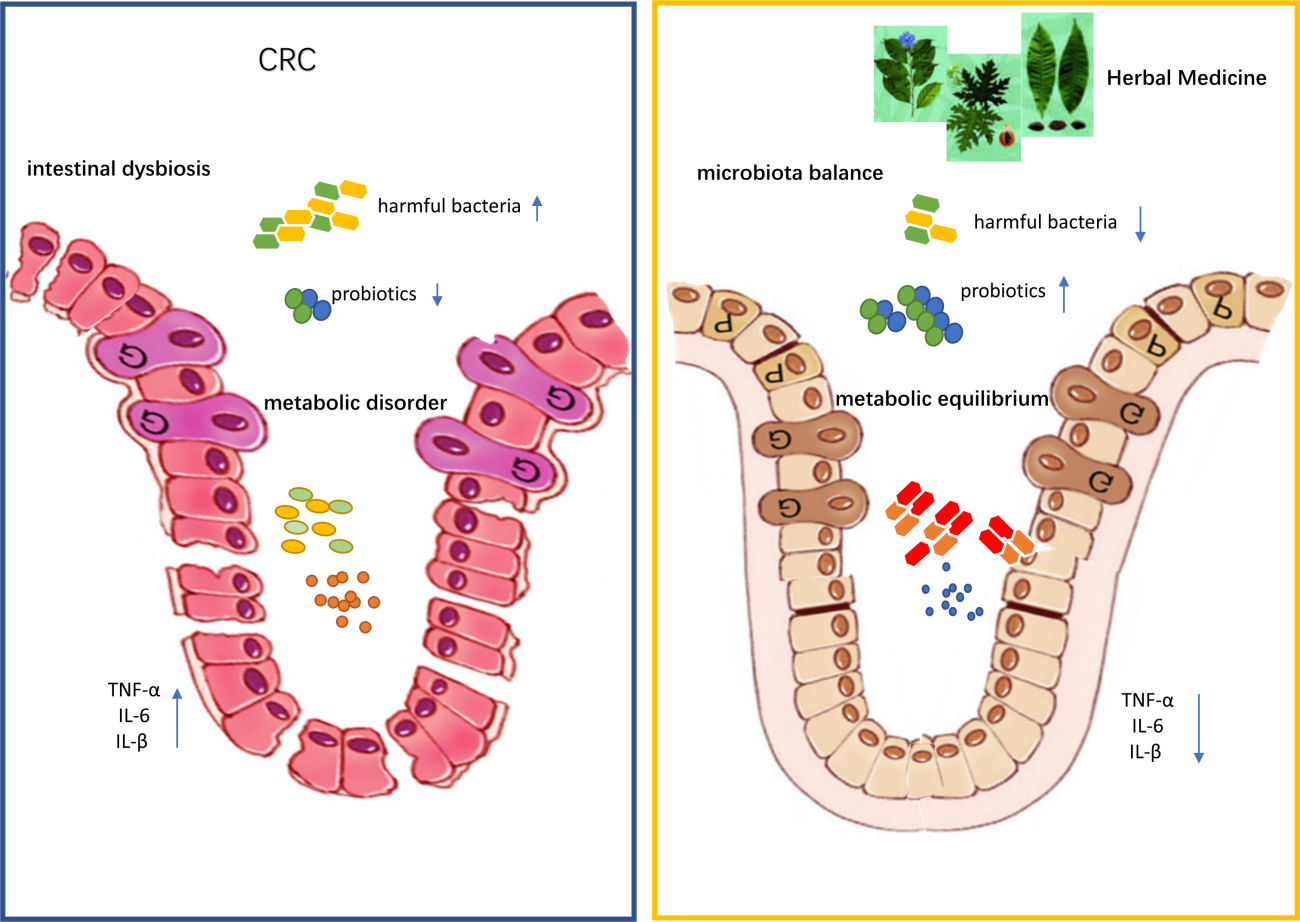
Traditional Chinese medicine improves colon cancer symptoms by regulating gut microbiota and its metabolites.

Taken together, the available data suggest that herbal medicine can modulate dysregulated microbiota during the development of CRC (**Table 1**). Herbal medicine exerted prebiotic-like effects and restored the intestinal flora diversity or mitigated gut dysbiosis compared with control groups (**Figure 1**).

**Table 1:** Effects of different Chinese medicine on CRC via regulating the gut microbiota.

Source	Main ingredients	Animal model	Chemical composition	Action on gut microbiota	Beneficial changes achieved
Coptis chinensis	isoquinoline alkaloid berberine	Apc ^min/+^ mice fed with an HFD	alkaloid	suppress *Akkermansia* and elevate some short-chain fatty acid (SCFA)-producing bacteria(*Lachnospiraceae*)	Attenuated the increase in bodyweight, reduced the tumor number and tumor load, reduced the expressions of cyclin D1 and β-catenin
Zizyphus jujuba	Muzao polysaccharides	AOM/DSS	polysaccharide	Increased the abundance of *Bifidobacterium*, *Bacteroides*, *Lactobacillus*, and *Clostridium_sp_K4410MGS-306*, decreased the abundance of *Firmicutes/Bacteroidetes*	Prevented CRC mouse colon shortening, decreased their mortality, reduced pro-inflammatory cytokines, increased the concentration of SCFAs
jujube fruit	jujube polysaccharides	AOM/DSS	polysaccharide	Increased the abundance of *Lactobacillaceae*, *Bacteroidaceae* and *Debaryomycetaceae*; decreased the abundance of *Herpotrichiellaceae*, *Enterobacteriaceae*, *Aspergillaceae* and *Lachnospiraceae*	Decreased the number and sizes of tumors
Poria cocos	Carboxymethylated pachyman	AOM/DSS	polysaccharide	Increased the proportion of *Bacteroidetes*, *Lactobacilli*, and butyric acid-producing and acetic acid-producing bacteria	Reversed intestinal shortening, alleviated 5-fluorouracil-induced colon injury; decreased the expression of NF-κB, p-p38 and Bax.
Curcumin	Curcuma longa plant	AOM/Il10^−/−^	polyphenol	Increased the relative abundance of *Lactobacillales*, and decreased *Coriobacterales* order	Increased survival, decreased colon weight/length ratio, eliminated tumor burden.
polysaccharides isolated from N. commune	Nostoc commune Vaucher	AOM/DSS	polysaccharide	Increased the number of the SCFA-producing genera, (*Butyricicoccus*, *Butyrivibrio* and *Butyricimonas*, *Lachnospiraceae UCG 001*, *Lachnospiraceae UCG 006*, and *Blautia*)	Decreased the number and sizes of tumors and reduced the incidence of intestinal tumors.
Isoliquiritigenin	licorice	AOM/DSS	flavonoids	reduced the abundance of *Escherichia* and *Enterococcus*, and increased the levels of butyrate-producing bacteria (*Butyricicoccus*, *Clostridium*, and *Ruminococcus*)	Decreased the incidence of cancer, decreased the levels of many cytokines (IL-6, IL-10, TNF-α, IL-1β)
Apiumgraveolens L.	Apigenin	AOM/DSS	flavonoids	Increased the relative abundance of *Firmicutes* and *Actinobacteria*	Decreased tumor morbidity
green tea	epigallocatechin gallate	AOM/DSS	polyphenol	Increased the population of *Bifidobacterium* and *Lactobacillus*	Reduces experimental colitis and inhibits the formation of aberrant crypt foci
black raspberry	anthocyanins	AOM/DSS	polyphenol	Increase the abundance of *Eubacterium rectale*, *Faecalibacterium prausnitzii* and *Lactobacillus*, decrease the abundance of *Desulfovibrio*. and *Enterococcus*	Downregulated the expression levels of DNMT31, DNMT3B and p-STAT3
Flos Sophorae Immaturus	Quercetin	AOM/DSS	flavonoids	Decreased the abundance of *Proteobacteria*, increases the relative abundance of *Actinobacteria*, *Firmicutes*, and *Bacteroides*	Cytoprotective effects in the intestinal epithelium and protection against colon tumorigenesis, reduce tumor numbers
Gynostemma pentaphyllum	Ginsenosides Rb3 and Rd	Apc^Min/+^ mice	triterpenes	Increased the population of *Bifidobacterium*, *Lactobacillus*, and *Bacteroides*; reduced the abundance of *A.muciniphila*, *Dysgonomonas* and *Helicobacter*	Reduced the size and the number of the polyps, promoted goblet and Paneth cells population, improved intestinal epithelial structure
Panax quinquefolius L.	ginsenosides	AOM/DSS	triterpenes	Decreased the levels of *Bacteroidetes* and *Verrucomicrobia*	Reduced tumor multiplicity, inhibited inflammatory cytokines, and increased the glutamine level
Gegen Qinlian decoction	Radix Puerariae, Scutellariae Radix,Coptidis Rhizoma, and liquorice	CT26 tumour	–	Increased the population of *Bacteroides_acidifaciens* and *Bacteroidales_S24-7_group*	Inhibited the growth of CT26 tumours
Yi-Yi-Fu-Zi-Bai-Jiang-San	Semen Coicis, monkshood, Herba Patriniae	Apc^Min/+^ mice	–	Increased the abundance of *Bifidobacterium* and *Prevotellaceae*; decreased the abundance of *Bacteroides*, *Lachnospiraceae*, *unclassified lachnospiraceae*	Suppresses intestinal tumorigenesis and expression of Ki67, PCNA, and reactivity to BrdU
Wu Mei Wan	Herba Hedyotidis, Scutellariae Barbatae Herba,Paridis Rhizoma et al.	AOM/DSS	–	decreased the abundance of *bacteroidales_s24-7_group*; increased the population of *Lachnospiraceae*	Decreased the expression of p65, IL-6, and p-STAT3, reduce DAI score, tumor formation, tumor volume, and grade of tumorigenesis

## Future perspectives

In recent decades, GM’s role in mediating the therapeutic efficacy of Chinese medicine has been uncovered. This review discusses and provides a comprehensive overview of the existing evidence and research progress. There is an increasing consensus that GM is closely related to the occurrence and development of CRC and represents a new drug target for treating CRC. The above studies overlap in their assertion that GM and its metabolism play a key role in CRC mediated by immune system stress, amino acid metabolism, protein expression, and gene regulation.

TCM plays an important role in the treatment of several diseases. TCM plants contain various classes of secondary plant metabolites, which are poorly absorbed in the upper GI tract due to their high polarity and molecular weight. Therefore, it is highly conceivable that they interact with the gut microbiome, thereby modulating GM and its metabolism. It has been documented that Chinese medicine regulates the composition of GM and metabolic activities through the GM to treat CRC. Certain compound classes, such as polysaccharides, have been shown to exert prebiotic effects. Terms such as flavobiotics have been used to refer to phytochemical constituents conferring health benefits on the host by positively influencing the gut microbiome. Moreover, some of the plant constituents can be metabolized by GM into pharmacologically active compounds and other postbiotics such as SCFAs, BAs, and polyamines metabolites that can either have a local effect in the gut or be absorbed by the epithelial cells and provide other health benefits to the host via different pathways. TCM can work synergistically with chemotherapeutic drugs to treat CRC, increase their efficacy, reduce their side effects and yield better curative effects.

Most of the existing studies focus on the efficacy of TCM in treating diseases and the regulation of intestinal flora, and lack of further experimental verification and in-depth discussion of the mechanism. In addition, the components of TCM are complex, so it is difficult to explore the molecular mechanism of its specific treatment of diseases. The separation and purification of the components of TCM may contribute to the exploration of the mechanism. Further research on how the Chinese medicine compound affects GM and its metabolism *in vivo* is warranted. Emphasis should be paid to herbal medicines to better understand the relationship between Chinese medicine and GM, providing the foothold for personalized GM management and treatment with TCM.

## Author contributions

FB and YT wrote an original draft, while ST and ZW reviewed the draft. All authors contributed to the article and approved the submitted version.
